# Supplementary material for: Characteristics and Trends of Workplace Violence towards Frontline Health Workers under Comprehensive Interventions in a Chinese Infectious Disease Hospital
Source: Healthcare (Basel). 2024 Sep 24;12(19):1911. doi: 10.3390/healthcare12191911 (PMC11475492; doi:10.3390/healthcare12191911)
Supplement: Supplementary file 1 [file healthcare-12-01911-s001.zip › healthcare-3162892-supplementary.pdf]

File S1:

Semi-structured interview questions about WPV in the Hospital.

Theme	No.	Questions
Incidents	1	What is the definition of WPV? Please give an example?
	2	Have you ever experienced WPV in the previous year? Please describe the most serious case.
Factors	3	What are the consequences of WPV?
	4	What is the main cause of physical violence in the workplace?
	5	What is the main cause of psychological violence in the workplace?
Measures	6	What measures has the hospital taken to address WPV?
	7	What is the most effective measure to combat WPV?
Actions during COVID-19	8	Did your hospital protect health workers free from WPV during COVID-19? What were the outcomes？
Plans	9	What else do you think your hospital should do to prevent and control WPV in the near future?

10	Based on your job responsibilities, what is your plan for addressing WPV?

File 2:

Consolidated criteria for reporting qualitative studies (COREQ) checklist[1]

Topic	Item No.	Guide Questions/Description	Reported on Page No.
**Domain 1: Research Team and Reflexivity**			
Personal Characteristics			
Interviewer/facilitator	1	Which author/s conducted the interview or focus group?	YH, FW, YL, JW, QL
Credentials	2	What were the researcher's credentials? e.g. PhD, MD	Title page
Occupation	3	What was their occupation at the time of the study?	PHD candidate/Master degree candidate
Gender	4	Was the researcher male or female?	female
Experience and training	5	What experience or training did the researcher have?	Master/University degree, practical experience
Relationship with Participants			
Relationship established	6	Was a relationship established prior to study commencement?	2.3.1 Interview data collection
Participant knowledge of the interviewer	7	What did the participants know about the researcher? e.g. personal goals, reasons for doing the research	2.3.1 Interview data collection
Interviewer characteristics	8	What characteristics were reported about the interviewer/facilitator? e.g. bias, assumptions, reasons and interests in the research topic	N/A
**Domain 2: Study Design**			
Theoretical Framework			
Methodological orientation and theory	9	What methodological orientation was stated to underpin the study? e.g. grounded theory, discourse analysis, ethnography, phenomenology, content analysis	2.3.3 Data coding and analysis
Participant selection			
Sampling	10	How were participants selected? e.g. purposive, convenience, consecutive, snowball	2.3.1 Interview data collection

Method of approach	11	How were participants approached? e.g. face-to-face, telephone, mail, email	2.3.1 Interview data collection
Sample size	12	How many participants were in the study?	2.3.2 Interviewee characteristics
Non-participation	13	How many people refused to participate or dropped out? Reasons?	None
Setting			
Setting of data collection	14	Where was the data collected? e.g. home, clinic, workplace	2.3.1 Interview data collection
Presence of non-participants	15	Was anyone else present besides the participants and researchers?	2.3.1 Interview data collection
Description of sample	16	What are the important characteristics of the sample? e.g. demographic data, date	2.3.2 Interviewee characteristics
Data Collection			
Interview guide	17	Were questions, prompts, guides provided by the authors? Was it pilot tested?	2.3.1 Interview data collection
Repeat interviews	18	Were repeat interviews carried out? If yes, how many?	No
Audio/visual recording	19	Did the research use audio or visual recording to collect the data?	2.3.3 Data coding and analysis
Field notes	20	Were field notes made during and/or after the interview or focus group	2.3.1 Interview data collection
Duration	21	What was the duration of the interviews or focus group?	2.3.1 Interview data collection
Data saturation	22	Was data saturation discussed?	2.3.1 Interview data collection
Transcripts returned	23	Were transcripts returned to participants for comment and/or correction?	NO; 2.3.1 Interview data collection

**Domain 3: Analysis and Findings**			
Data Analysis			
Number of data coders	24	How many data coders coded the data?	2.3.3 Data coding and analysis
Description of the coding tree	25	Did authors provide a description of the coding tree?	2.3.3 Data coding and analysis
Derivation of themes	26	Were themes identified in advance or derived from the data?	2.3.3 Data coding and analysis
Software	27	What software, if applicable, was used to manage the data?	2.3.3 Data coding and analysis
Participant checking	28	Did participants provide feedback on the findings?	No
Reporting			
Quotations presented	29	Were participant quotations presented to illustrate the themes/findings? Was each quotation identified? e.g. participant number	Results, all paragraphs
Data and findings consistent	30	Was there consistency between the data presented and the findings?	Discussion
Clarity of major themes	31	Were major themes clearly presented in the findings?	Results
Clarity of minor themes	32	Is there a description of diverse cases or discussion of minor themes?	Results

1. TongA, Sainsbury P, Craig J. Consolidated criteria for reporting qualitative research (COREQ): a 32 -item checklist for interviews and focus groups. IntJ Qual Health Care 2007; 19(6):349 - 357

File 3:
Characteristics and frequency distributions for workplace violence among 720 HWs in 2021.

Characteristic	N	%	Physical violence			Verbal abuse			Bullying/Mobbing			Sexual harassment			Ethnic discrimination		
			n	%	P- value	n	%	P- value	n	%	P- value	n	%	P- value	n	%	P-value
Gender					0.19			0.36			0.34			0.29			1.00
Female	533	74.03	15	2.81		179	33.58		42	7.88		10	1.88		7	1.31	
Male	187	25.97	9	4.81		56	29.95		19	10.16		6	3.21		2	1.07	
Age（y）					0.75			0.10			0.11			0.40			0.81
<35	384	53.33	11	2.86		112	29.17		25	6.51		11	2.86		4	1.04	
35~44	226	31.39	9	3.98		84	37.17		23	10.18		4	1.77		3	1.33	
45~	110	15.28	4	3.64		39	35.45		13	11.82		1	0.91		2	1.82	
Ethnic groups					0.35			0.63			0.68			0.80			0.19
Han	337	46.81	9	2.67		113	33.53		27	8.01		7	2.08		2	0.59	
Zhuang and others	383	53.19	15	3.92		122	31.85		34	8.88		9	2.35		7	1.83	
Marital status					0.63			0.37			0.95			0.38			0.73
Single	213	29.58	8	3.76		64	30.05		17	7.98		7	3.29		3	1.41	

Married	484	67.22	16	3.31		161	33.26		42	8.68		9	1.86		6	1.24	
others	23	3.19	0	0.00		10	43.48		2	8.70		0	0.00		0	0.00	
Educational level					0.47			0.93			0.40			0.65			0.45
Junior college or/and below	168	23.33	8	4.76		53	31.55		10	5.95		3	1.79		3	1.79	
bachelor degree	502	69.72	15	2.99		166	33.07		46	9.16		11	2.19		6	1.20	
Master degree or/and above	50	6.94	1	2.00		16	32.00		5	10.00		2	4.00		0	0.00	
Occupation					0.11			<0.01			0.03			0.97			0.17
Technical support and administration	195	27.08	2	1.03		37	18.97		8	4.10		4	2.05		1	0.51	
Doctor	340	47.22	14	4.12		127	37.35		32	9.41		8	2.35		7	2.06	
Nurse	185	25.69	8	4.32		71	38.38		21	11.35		4	2.16		1	0.54	
Professional title					0.49			<0.01			<0.01			0.88			0.91
Junior or/and below	356	49.44	9	2.53		103	28.93		18	5.06		8	2.25		4	1.12	
Middle	243	33.75	10	4.12		75	30.86		25	10.29		6	2.47		3	1.23	
Senior	121	16.81	5	4.13		57	47.11		18	14.88		2	1.65		2	1.65	
Work tenure					0.14			0.01			0.01			0.42			0.42
<10	393	54.68	10	2.54		108	27.48		22	5.60		9	2.29		3	0.76	

10~19	201	27.92	11	5.47		79	39.30		22	10.95		6	2.99		4	1.99	
20~	126	17.50	3	2.38		48	38.10		17	13.49		1	0.79		2	1.59	
Contract status					0.21			0.01			0.01			0.51			0.74
Permanent	329	45.69	14	4.26		134	40.73		38	11.55		6	1.82		5	1.52	
Temporary	391	54.31	10	2.56		101	25.83		23	5.88		10	2.56		4	1.02	
Salary (yuan/M)					0.14			0.01			0.51			0.07			0.68
<2 000	39	5.42	2	5.13		4	10.26		2	5.13		2	5.13		1	2.56	
2 000~	167	23.19	4	2.40		53	31.74		11	6.59		1	0.60		1	0.60	
4 000~	238	33.06	4	1.68		76	31.93		20	8.40		3	1.26		4	1.68	
6 000~	276	38.33	14	5.07		102	36.96		28	10.14		10	3.62		3	1.09	
Department					0.32			<0.01			0.01			0.83			0.02
Technical support and Administration department	221	30.69	4	1.81		53	23.98		12	5.43		4	1.81		0	0.00	
Outpatient and emergency	144	20.00	6	4.17		61	42.36		21	14.58		4	2.78		4	2.78	
Ward and other	355	49.31	14	3.94		121	34.08		28	7.89		8	2.25		5	1.41	
Work in shifts					0.68			0.42			0.55			0.04			0.70
No	144	20.00	4	2.78		43	29.86		14	9.72		0	0.00		1	0.69	

Yes	576	80.00	20	3.47		192	33.33		47	8.16		16	2.78		8	1.39	
Work in nights					0.04			0.19			0.64			0.09			0.29
No	232	32.22	3	1.29		68	29.31		18	7.76		2	0.86		1	0.43	
Yes	488	67.78	21	4.30		167	34.22		43	8.81		14	2.87		8	1.64	
Have direct physical contact/ interaction with patients					0.23			<0.01			0.07			0.47			0.61
No	87	12.08	1	1.15		6	6.90		3	3.45		1	1.15		0	0.00	
Yes	633	87.92	23	3.63		229	36.18		58	9.16		15	2.37		9	1.42	
Worry about workplace violence					0.75			<0.01			0.01			0.49			0.22
No	138	19.17	4	2.90		19	13.77		4	2.90		2	1.45		0	0.00	
Yes	582	80.83	20	3.44		216	37.11		57	9.79		14	2.41		9	1.55	
Total	720	100.00	24	3.33		235	32.63		61	8.47	0.34	16	2.22		9	1.25	

File 4:

Codebook and verbatim supporting each theme/subtheme

Themes	Subthemes	Codes	Verbatim
Incidents	definition	physical violence and psychological violence	"WPV refers to incidents that pose a threat to personal safety within a hospital. Violence can be definitely classified as physical attacks or verbal aggression."(Doctor, P3DOC)<br>"The main form of violence is verbal violence. Within the hospital, there may be physical violence and verbal violence, but we haven't experienced any incidents of Ethnic discrimination. Communication with colleagues from other departments can also be challenging (of WPV). "(Doctor, P5DOC)
	experience	internal violence and eternal violence events	"Verbal violence almost happened every day. The most extreme case was probably in last year, a liver cancer patient unsatisfied with the long waiting time. He then verbally abused the nurses responsible for maintaining order. " (Doctor, P4DOC)<br>"A surgeon encountered a drunken injury patient without a clear consciousness, my colleague wanted to suture his wound, so he needed to hold him in a restrained position, but he refused and then hit (my colleague)...As for harassment, my colleagues have experienced more explicit and offensive remarks." (Nurse, P4NUR)
Factors	consequences	negative impacts on individual<br>negative impacts on organization<br>negative impacts on family	"For example, I experienced violence in work, and complained about it when I was home. My family members worried about what happened to me, sometimes even more than I did. " (Nurse, P3NUR)<br>"WPV affected my mood and also disrupted my work. When I provided discharge instructions to patients, I needed to concentration on my tasks, someone suddenly yelled at me, it distracted my attention and made me difficult to stay calm." (Nurse, P8NUR)

	Causes of physical violence	unmet expectation of treatment<br>perpetrators' personal factors<br>social factors<br>workplace factors<br>victims' personal factors	"The first factor (of physical violence) was the general public's understanding of nurses and doctors. The second factor may be the psychological resilience of individual HW...." (Nurse, P2NUR)<br>"First and foremost, it was definitely due to our own personal reasons. Our attitude and way of speaking may also be affected by the busy (work), we had an overwhelming number of patients in our ward, which made us feel stressed and irritable." (Nurse, P5NUR)<br>"The waiting time (for outpatient) in our facility was relatively long. When we tried to increase the staff number, the limited space restricted our ability to do so." (Nurse, P9NUR)
	Causes of psychological violence	perpetrators' personal factors<br>unmet expectation of treatment<br>workplace factors<br>victims' personal factors<br>social factors	"Some family members may demand a clear diagnosis of TB right from the beginning, however, based on the current available resources and conditions, it was difficult to promise a 100% for this." (Doctor, P7DOC)<br>"In reality, the underlying reason (of PWV) was often financial constraints (for patients). Patients may spend a significant amount of money, hoping for better results. However, when their expectations were not met under financial difficulties, it can lead to frustration and dissatisfaction." (Nurse, P8NUR)
Measures	current measures	environmental intervention<br>organizational intervention<br>individual intervention<br>other measures	"If there is an urgent situation, we can push the button of the one-touch alarm system to alert the security personnel who will promptly respond and resolve the issue. Additionally, strict access control measures are in place. Patients are unable to enter restricted areas, effectively creating a separation between our workspace and the patients' areas." (Doctor, P2DOC)<br>"In terms of shift scheduling, we made efforts to lighten our workload by proper shift patterns and staff matching reasonably, this helped alleviate the emotional strain caused by busy workloads. " (Nurse, P4NUR)

Actions during COVID-19	the most effective measures	individual-level measures<br>hospital-level measures	“One of the effective approaches was to reduce the workload of healthcare professionals.” (Doctor, P7DOC)
		societal-level measures	“Installing metal detection systems at the entrance of the hospital for timely detection sharp objects or weapons... strengthening the security visibility in the hospital helps ensure a safer environment.” (Doctor, P9DOC)<br>“According to my decades of work experience...an effective measure was to timely split tasks among more staff when the workload was getting higher.” (Nurse, P4NUR)
	approaches	environmental intervention<br>organizational intervention	“In the past, we had difficulty to identify if the individual was HWs at the entrance, but now the access system with facial recognition technology works.” (Doctor, P1DOC)
		individual-level intervention	“From my observation, there are more security personnel now than before, but I'm not sure about the exact increased number. However, the intensity of patrols has significantly increased.” (Doctor, P2DOC)
	outcomes	ensuring personal safety<br>relieving the tension of the doctor-patient relationship	“Since the outbreak of COVID-19, we set many good examples of our dedication to the public, in my communication with patients' families today, I can feel their deep sense of respect.” (Doctor, P1DOC)
		deterring the perpetrators	“I believe that our hospital was relatively good in this aspect, such as the competency of the security personnel and their prompt response. When we are on duty, we feel really safe.” (Doctor, P4DOC)
Plans	hospital improvement	strengthening organizational intervention<br>improving the working environment<br>enhancing individuals’ coping abilities	“One (priority) is to improve the process and environment of the medical treatment, as well as the system relevant to WPV for their implementation.” (Doctor, P6DOC)

	emphasizing post-incident support	“There is a lack of a reporting mechanism for violence. Currently, only incidents involving blood-borne pathogens or needle-stick injuries were reported, but no specific reporting policy in place for verbal violence.” (Doctor, P8DOC)<br>“Once the WPV occurs, it is important for the hospital management not to blindly criticize our own staff.” (Doctor, P9DOC)
	enhancing personal coping abilities<br>strengthening patient-centered care	“I have been in the medical field for over 10 years, and I always believe that, if we approach problems from the perspective of the patient and understand what they need, help patients understand our medical actions by using various channels, it can make a significant difference.” (Doctor, P1DOC)
Individuals improvement	seeking external support	“First and foremost, training is essential. My team must prioritize patient-centered care and understand the patients' perspectives. Secondly, when facing unreasonable and difficult patient, it is important to know how to actively report, calm handling, and protect ourselves.” (Doctor, P3DOC)
